# Maternal morbidity and neonatal survival after periviable PPROM: the impact of latency duration

**DOI:** 10.1186/s12884-026-09759-x

**Published:** 2026-08-07

**Authors:** Selma Atiye Kolcu, Habibe Ayvacı Taşan

**Affiliations:** 1 Present Address: Department of Gynecology and Obstetrics, Istanbul Training And Research Hospital, Cerrahpaşa, General Abdurrahman Nafiz Gürman St. No:24, Istanbul, Fatih 34098 Türkiye; 2 Department of Obstetrics and Gynecology, Zeynep Kamil Women and Children’s Diseases Training and Research Hospital, Istanbul, Türkiye

**Keywords:** Fetal membranes, Fetal viability, Maternal morbidity, Pregnancy, Second trimester, Premature rupture, Termination of pregnancy

## Abstract

**Background:**

Periviable preterm premature rupture of membranes (PPROM) is a rare (< 1%), critical obstetric complication associated with profound maternal and neonatal morbidity. Management involves a delicate trade-off between optimizing neonatal survival via pregnancy prolongation and mitigating maternal risks like chorioamnionitis, sepsis, and placental abruption. While expectant management is the standard to reach viability, research has predominantly focused on neonatal outcomes. Consequently, the potential for progressive, daily accumulation of maternal risk during latency remains insufficiently characterized. This study aims to identify maternal morbidity and risk factors, specifically evaluating the impact of latency duration on both safety and survival to provide objective data for clinical counseling and shared decision-making.

**Methods:**

This retrospective cohort study analyzed 212 cases of PPROM diagnosed before 24 + 6 weeks’ gestation. Patients were classified by initial management into expectant management (*n* = 84) or termination of pregnancy (*n* = 128) groups. The primary outcome was composite maternal morbidity (CMM). To address confounding by indication, propensity-score matching was performed within the sub-23-week subcohort. Maternal complication accrual over the latency interval was evaluated using a competing-risks framework. Factors associated with neonatal survival to discharge were analyzed using Firth-penalized multivariable logistic regression to account for small-sample bias.

**Results:**

In the propensity-score-matched cohort, expectant management was independently associated with a significant increase in CMM (matched OR 3.22; 95% CI 1.31–7.92; *p* = 0.011). Overall CMM rates were 32.1% in the expectant group and 11.7% in the termination group. Maternal temperature > 37 °C at admission was the strongest independent predictor of maternal morbidity across the entire cohort (OR 4.37; 95% CI 1.59–11.99; *p* = 0.005). Within the expectant management arm, each additional day of latency was not associated with a progressive increase in daily maternal risk (*p* = 0.299). Conversely, each day of pregnancy prolongation was significantly associated with an increased probability of neonatal survival to discharge (adjusted OR 1.058 per day; 95% CI 1.027–1.102; *p* < 0.001). Among live-born infants managed expectantly, the survival-to-discharge rate was 63.6%.

**Conclusion:**

Expectant management of periviable PPROM is associated with a more than three-fold increase in maternal morbidity compared to termination; however, this risk does not escalate progressively with each additional day of latency. In contrast, every day of pregnancy prolongation significantly enhances the probability of neonatal survival to discharge. These findings indicate that in the absence of acute maternal complications and under vigilant clinical supervision, expectant management represents a rational and viable strategy to optimize neonatal outcomes without a compounding daily risk profile for the mother.

**Supplementary Information:**

The online version contains supplementary material available at https://doi.org/10.1186/s12884-026-09759-x.

## Introduction

Premature rupture of membranes (PROM) complicates approximately 5 to 7% of all pregnancies. Prior to 24 weeks of gestation, the incidence is lower (0.3–0.4%), but early-onset PROM carries substantially higher maternal and neonatal morbidity and mortality [1, 2]. In pregnancies complicated by preterm premature rupture of membranes (PPROM) managed conservatively, approximately 50% of women deliver within one week of membrane rupture [3, 4]. Notably, as the gestational age at the time of rupture decreases, the latency period between membrane rupture and delivery correspondingly increases. In cases of membrane rupture occurring before 24 weeks of gestation, reported latency periods range from 13 to 80 days [5, 6].

Periviability is generally defined as the earliest stage of fetal maturity and is associated with a high incidence of both short- and long-term morbidity and mortality. Although there is no universal consensus, the threshold of 25 weeks of gestation is frequently used to define viability [7]. Spontaneous cessation of amniotic fluid leakage and subsequent reaccumulation of fluid, often termed 'resealing,' remains an infrequent but recognized clinical event in periviable PPROM [8, 9]. While such cases may achieve significantly longer latency, spontaneous resealing remains an exceptional occurrence in the natural history of spontaneous PPROM [8, 10]. This clinical course is distinct from iatrogenic PPROM (e.g., following genetic amniocentesis), where membrane resealing is observed more frequently and is linked to a substantially better prognosis, often resulting in favorable neonatal outcomes [8, 9].

Traditional counseling emphasizes that termination reduces maternal risk compared to expectant management (morbidity: 20%–33% vs. 40%–60.2%) [9, 11, 12]. However, PPROM inherently imposes a significant baseline risk that is not entirely eliminated even if termination is chosen [13]. Evidence regarding the specific impact of latency duration on maternal outcomes remains limited and heterogeneous, as research frequently prioritizes neonatal survival over the characterization of maternal risk. Given this uncertainty, we hypothesized that although expectant management carries a higher baseline morbidity risk than termination, the duration of pregnancy prolongation does not lead to a progressive increase in maternal complications. Furthermore, we hypothesized that expectant management significantly improves the odds of neonatal survival to discharge.

## Materials and Methods

This single-center, retrospective, prognostic cohort study was conducted at a tertiary referral center between March 1, 2013, and March 1, 2019. Within the designated study period, we identified all 221 consecutive pregnant women (aged 15–45 years) who were diagnosed with PPROM at ≤ 24 weeks and 6 days of gestation. Pregnancies with known fetal anomalies, multiple pregnancies other than twins, medical conditions contraindicating expectant management, deliveries or pregnancy losses occurring within 24 h after membrane rupture, and pregnancies delivered at other hospitals were excluded from the study. The study protocol was approved by the Institutional Review Board (Date: 20.03.2019, Number: 34).

Data were collected from patients’ electronic records and hospital archive files. For babies born at our hospital but subsequently referred to neonatal intensive care units in other hospitals, data were obtained from the relevant hospitals upon presentation of the ethics committee approval.

The diagnosis of membrane rupture was considered accurate if active amniotic fluid leakage was observed on sterile speculum examination, or pooling in the posterior fornix was detected, or if confirmatory tests such as PAMG-1 determination were used.

Gestational age (GA) was confirmed using the last menstrual period and first-trimester ultrasound. For expectantly managed pregnancies, GA at PPROM, GA at delivery, and latency duration (defined as the interval from PPROM to delivery or termination) were recorded. Amniotic fluid volume was monitored from admission until delivery; oligohydramnios was defined as a deepest vertical pocket (DVP) < 2 cm or a four-quadrant amniotic fluid index (AFI) < 5 cm, while anhydramnios was defined as DVP < 1 cm. Follow-up included weekly ultrasound evaluations and non-stress tests (NST) beyond the viability threshold.

Comprehensive data were collected on maternal demographics, obstetric history and current pregnancy conditions (smoking, leiomyoma, intrauterine devices, cervical procedures, hypertensive disorders, diabetes, and antepartum hemorrhage). Diagnostic markers at PPROM, including the BISHOP score, cervical length, and concomitant vaginal bleeding, were also documented.

Cases diagnosed < 23 weeks’ gestation were allocated to expectant management or termination based on patient preference; all cases ≥ 23 weeks were managed expectantly. The medical protocol consisted of a 1-g dose of azithromycin and a 7-day course of ampicillin. For pregnancies ≥ 23 weeks, two 12-mg intramuscular doses of betamethasone were administered 24 h apart, with a rescue course planned after 14 days if delivery was not imminent and GA was < 34 weeks. Maternal inflammatory markers (temperature, leukocytes, and CRP) and perinatal outcomes (birth weight, Apgar scores, resuscitation needs, and discharge status) were analyzed.

Clinical chorioamnionitis was diagnosed based on the presence of maternal fever (≥ 38 °C) in association with at least two of the following criteria: maternal or fetal tachycardia, uterine tenderness, purulent or foul-smelling vaginal discharge, and elevated CRP or leukocyte levels. Maternal sepsis was defined as maternal fever (≥ 38 °C), positive blood culture, and evidence of organ dysfunction (hypotension, oliguria, elevated creatinine, disseminated intravascular coagulation (DIC), altered consciousness, or respiratory failure).

Maternal and obstetric complications, including placental abruption, cord prolapse, uterine atony, postpartum fever, retained placenta (requiring manual removal or revision curettage), postpartum laparotomy or hysterectomy, wound infection, the need for blood transfusion were recorded.

Composite maternal morbidity was defined as the presence of one or more of the following: clinical chorioamnionitis, postpartum endometritis, sepsis, unplanned postpartum surgical procedures (laparoscopy or laparotomy), injuries requiring repair, hysterectomy, non-delivery hysterectomy, uterine rupture, blood loss > 1000 mL, need for transfusion, acute renal failure, venous thromboembolism, pulmonary embolism, intensive care unit (ICU) admission, or hospital readmission within six weeks postpartum [1]. The single maternal death is retained within the composite maternal morbidity outcome; this case also carried transfusion and sepsis, so it contributes to the composite through coded components.

Postpartum hospitalization duration was recorded. One patient was discharged on postpartum day 2 but was readmitted due to active vaginal bleeding and underwent hysterectomy. The total length of both admissions was recorded as the postpartum hospitalization period.

### Statistical analysis

Continuous variables were summarized as mean ± standard deviation or median with interquartile range according to the Shapiro–Wilk test of normality and compared between management groups with the Student t-test or the Mann–Whitney U test. Categorical variables were summarized as counts with percentages and compared with the Pearson chi-square or Fisher exact test. For each comparison an effect size with a 95% confidence interval was reported using the measure appropriate to the variable type: the Hodges–Lehmann median difference with rank-biserial correlation for skewed continuous variables, the unadjusted odds ratio with the risk difference for binary variables, and Cramér's V for categorical variables. A two-sided p value of 0.05 or less was considered statistically significant.

Because expectant management was not randomly allocated, confounding by indication was addressed with propensity-score matching. The analysis was restricted to the subcohort below 23 weeks of gestation, within which the two management strategies overlapped in gestational age; this restriction was imposed to satisfy the positivity (common support) assumption. The target estimand was the average treatment effect on the treated, with expectant management treated as the exposed condition and termination as the control pool, consistent with one-to-one nearest-neighbor matching. The propensity model included maternal age, gestational age at admission, multiple gestation, assisted conception, and C-reactive protein. Missing C-reactive protein values were handled by multiple imputation using chained Eqs. (20 imputed sets, outcome included), and matching was performed within each imputed set with estimates pooled by Rubin's rules. Matching used a caliper of 0.2 times the standard deviation of the logit propensity score, without replacement; covariate balance was assessed by standardized mean differences rather than by significance tests, with an absolute standardized mean difference below 0.10 regarded as adequate. The matched effect of expectant management on composite maternal morbidity was estimated by Firth penalized conditional logistic regression, and the absolute risk difference was obtained by paired bootstrap resampling. Because the propensity score can balance only measured confounders, the analysis rests on the assumption of strong ignorability; residual confounding from uncoded factors such as the clinician's judgment of case severity cannot be excluded, and a proportion of expectant-management pregnancies fell outside common support and remained unmatched, which constrains the generalizability of the matched estimate.

Regression models used Firth penalized likelihood rather than maximum-likelihood estimation or stepwise selection, because the number of events per candidate variable was low and complete or quasi-complete separation was present in several predictors. Penalized profile-likelihood confidence intervals were reported, and the number of predictors was kept within the limit implied by the event count. This approach was applied to the whole-cohort model for composite maternal morbidity and to the neonatal model for survival to discharge.

The accrual of maternal complications over the latency interval was analyzed in a competing-risks framework, with latency as the time axis and complication-free delivery treated as the competing event. Cumulative incidence functions were estimated for the composite outcome and for its individual components, and subdistribution hazard ratios were obtained from Fine–Gray models with cause-specific Cox models reported alongside. Because the exact timing of each complication was not recorded, the time-to-event results are interpreted as the pattern of accrual over latency rather than as precise event times, and this is acknowledged as a limitation.

In the neonatal model, gestational age at birth and birth weight were not entered as adjustment covariates for the effect of latency, because they lie on the causal pathway between latency and neonatal survival and their inclusion would induce overadjustment. Latency was therefore modeled as the primary predictor of survival to discharge, with gestational age and birth weight examined only descriptively.

For families of related secondary outcomes, p values were adjusted for multiple comparisons by the Benjamini–Hochberg false-discovery-rate procedure, and both raw and adjusted values were reported so that no association was concealed. Analyses were performed in Python 3.11 (pandas, numpy, scipy, statsmodels) and R 4.5 (mice, MatchIt, logistf, cmprsk, survival, boot), with a fixed random seed to ensure reproducibility. Comparable descriptive procedures are available in jamovi (version 2.7.37) and JASP (version 0.98.1).

## Results

**Fig. 1:**
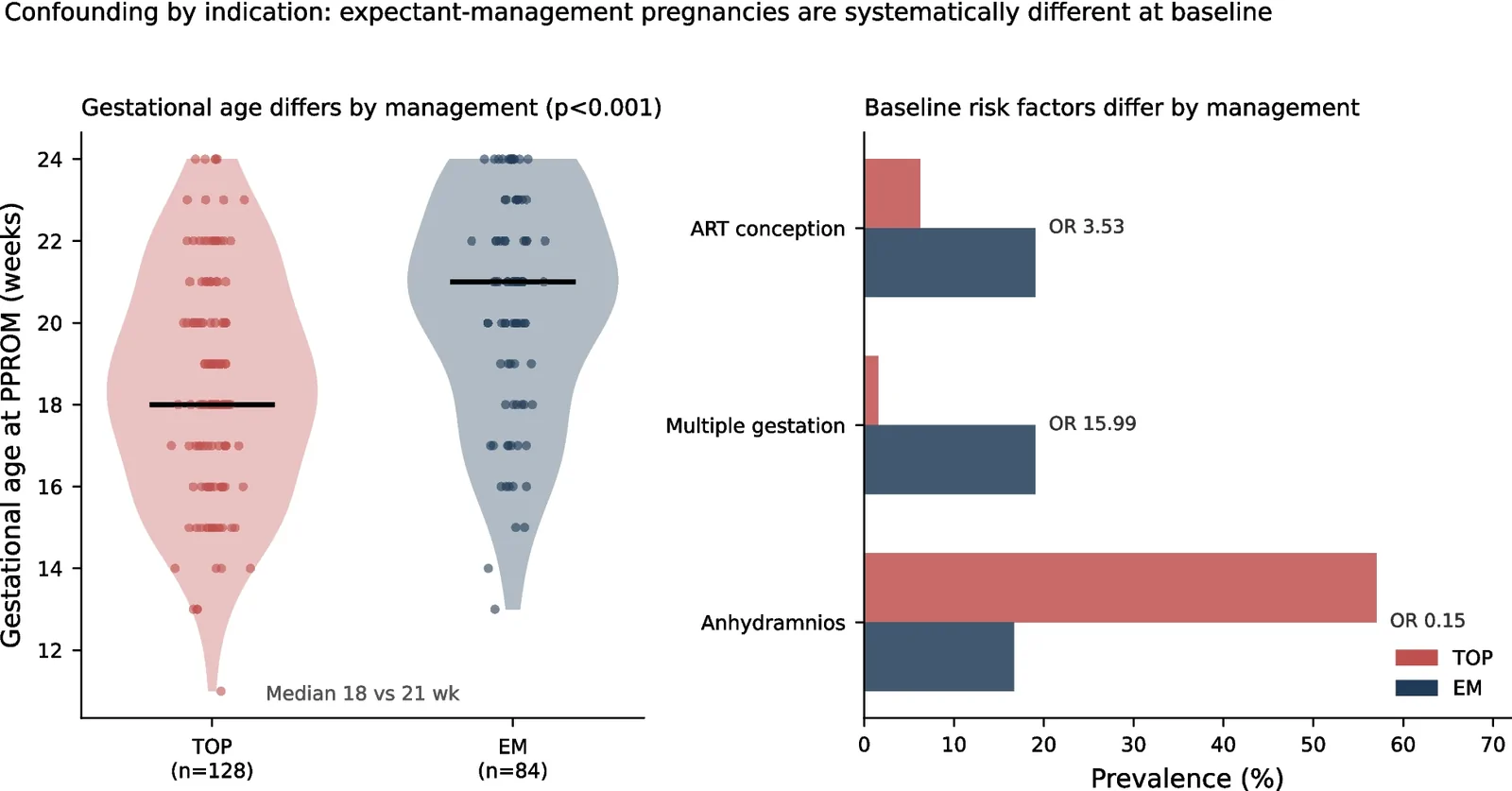
Baseline differences between termination and expectant-management groups (confounding by indication). Left: distribution of gestational age at PPROM by initial management (termination, red; expectant management, blue); violins show the density, points the individual pregnancies, and horizontal bars the group medians (18 vs 21 weeks, p < 0.001). Right: prevalence of key baseline risk factors by group, with unadjusted odds ratios annotated. Expectant-management pregnancies were systematically more advanced in gestation and differed in anhydramnios, multiple gestation, and assisted conception, motivating the propensity-score-matched and penalized analyses. ART assisted reproductive technology; PPROM, preterm premature rupture of membranes; TOP, termination of pregnancy; EM, expectant management

During the study period, a total of 45,118 deliveries were performed. During the study period, all consecutive pregnancies meeting the eligibility criteria were reviewed, and 221 cases of periviable preterm premature rupture of membranes (PPROM) were identified. Of 212 pregnancies with periviable preterm premature rupture of membranes, 128 (60.4%) underwent termination and 84 (39.6%) were managed expectantly. The two groups differed at baseline: expectant-management pregnancies were more advanced at membrane rupture (median gestational age 21 versus 18 weeks, *p* < 0.001) and had higher proportions of multiple gestation and assisted conception, whereas anhydramnios was more frequent in the termination group (Table 1,Fig. 1). These systematic baseline differences indicate confounding by indication and motivated the propensity-score-matched analysis.

**Table 1:** Baseline maternal, obstetric, and PPROM characteristics by initial management (TOP vs EM)

Variables	TOP (*n* = 128)	EM (*n* = 84)	p	Effect size (95% CI)
Maternal age (years), mean ± SD	30.3 ± 6.2	30.4 ± 5.8	0.984	—
Body-mass index (kg/m^2^), median [IQR]	26.5 [23.2–30.0]	26.5 [23.1–30.2]	0.839	—
Gestational age at PPROM (wk), median [IQR]	18 [16–20]	21 [18–22]	** < 0.001**	HL − 2.0 [− 3.0, − 1.0]; r = − 0.37
Latency (days), median [IQR]	3 [1–7]	30.5 [7–61]	** < 0.001**	HL − 20.0 [− 27, − 14]; r = − 0.55
Admission CRP (mg/L), median [IQR]	1.3 [0.7–3.3]	0.9 [0.5–1.8]	**0.003**	HL − 0.4 [− 0.8, − 0.15]; r = 0.27
Admission WBC (× 10^3^/µL), median [IQR]	12.1 [10.2–14.8]	11.7 [9.9–13.6]	0.240	r = 0.08
Anhydramnios, n (%)	73 (57.0)	14 (16.7)	** < 0.001**	OR 0.15 (0.08–0.30)
Multiple gestation, n (%)	2 (1.6)	17 (20.2)	** < 0.001**	OR 15.99 (3.59–71.27)
ART conception, n (%)	8 (6.2)	16 (19.0)	**0.004**	OR 3.53 (1.44–8.64)
Amniotic fluid category	Anhydramnios 73 (57.0); Oligohydramnios 32 (25.0); Normal 23 (18.0)	Anhydramnios 14 (16.7); Oligohydramnios 28 (33.3); Normal 42 (50.0)	** < 0.001**	Cramér V 0.43 (0.32–0.55)
Antepartum bleeding, n (%)	0 (0.0)	7 (8.3)	**0.001**	OR 24.87 (Haldane)†
Mode of delivery type	Spontaneous 5 (3.9); Induction 122 (95.3); Cesarean 1 (0.8)	Spontaneous 20 (23.8); Induction 16 (19.0); Cesarean 48 (57.1)	** < 0.001**	Cramér V 0.79

Values are median [IQR] or n (%). Effect sizes: Hodges–Lehmann (HL) median difference with rank-biserial r; unadjusted odds ratio (OR); Cramér V for categorical variables. Significance by Mann–Whitney U, chi-square or Fisher exact test. †TOP group had zero antepartum-bleeding events; Haldane correction applied, so the OR is unstable and directional only. Dashes denote cells omitted here for brevity in the analytic summary; full descriptive values appear in Table 3. Maternal age and body-mass index did not differ between groups. Multiple-gestation count reflects the source data (17 EM; 20.2%)

Bold *p*-values indicate statistically significant results (*p*< 0.05)

Maternal morbidity and its components by management group are shown in Table 2. Composite maternal morbidity, defined following the revised Sklar criteria, occurred in 27 of 84 expectant-management pregnancies (32.1%) and 15 of 128 termination pregnancies (11.7%). Clinical, laboratory, and ultrasonographic findings at admission are summarized in Table 3.

**Table 2:** Maternal morbidity and mortality by initial management (TOP vs EM)

Outcomes	TOP (*n* = 128), *n* (%)	EM (*n* = 84), *n* (%)	*p*
Composite maternal morbidity (Sklar)	15 (11.7)	27 (32.1)	** < 0.001**
Clinical chorioamnionitis	11 (8.6)	21 (25.0)	—
Postpartum hemorrhage (uterine atony)	3 (2.3)	4 (4.8)	—
Maternal sepsis	0 (0.0)	1 (1.2)	—
Manual removal of placenta	1 (0.8)	1 (1.2)	—
Unplanned laparotomy	2 (1.6)	1 (1.2)	—
Hysterectomy	1 (0.8)	1 (1.2)	—
Blood-product transfusion	4 (3.1)	7 (8.3)	—
Endometritis	0 (0.0)	0 (0.0)	—
Obstetric complications (reported separately)			
Placental abruption	2 (1.6)	7 (8.3)	—
Umbilical cord prolapse (excluded from composite)	4 (3.1)	2 (2.3)	—
Maternal death (reported separately)	0 (0.0)	1 (1.2)	—

Values are n (%). The composite maternal morbidity summary follows the revised Sklar criteria (umbilical cord prolapse excluded per reviewer request; total 42 events, TOP 15 / EM 27). Component rows are raw counts and may co-occur within a patient, so they need not sum to the composite. One maternal death (expectant management) co-occurred with other composite components and is retained within the composite while also reported separately. Dilation and curettage (procedural, TOP 101 / EM 16) is a management step rather than a morbidity outcome and is not part of the composite. Significance by chi-square or Fisher exact test

Bold *p-values* indicate statistically significant results (*p*< 0.05)

**Table 3:** Clinical, laboratory, and ultrasonographic findings at admission by initial management (TOP vs EM)

Variables	TOP (*n* = 128)	EM (*n* = 84)	*p*
WBC count at admission (× 10^3^/µL)	12.1 [10.2–14.8]	11.7 [9.9–13.6]	0.240
AFI at admission (mm)	42.5 [31.2–60.0]	62.5 [39.5–100.0]	**0.007**
CRP at admission (mg/L)	1.3 [0.7–3.3]	0.9 [0.5–1.8]	**0.003**
Maternal temperature at admission (°C)	36.5 [36.3–36.8]	36.4 [36.1–36.6]	**0.004**
Bishop score at admission	1.0 [1.0–2.0]	1.0 [1.0–2.0]	0.131
Low-grade temperature elevation (> 37 °C), n (%)	14 (11.0)	7 (8.3)	0.641
Anhydramnios at admission, n (%)	73 (57.0)	14 (16.7)	** < 0.001**

Continuous variables are median [IQR], compared by Mann–Whitney U test; categorical variables are n (%), compared by chi-square or Fisher exact test. WBC, white blood cell count; AFI, amniotic fluid index; CRP, C-reactive protein. Amniotic fluid index is reverse-coded such that anhydramnios denotes the most severe category

Bold *p*-values indicate statistically significant results (*p*< 0.05)

**Fig. 2:**
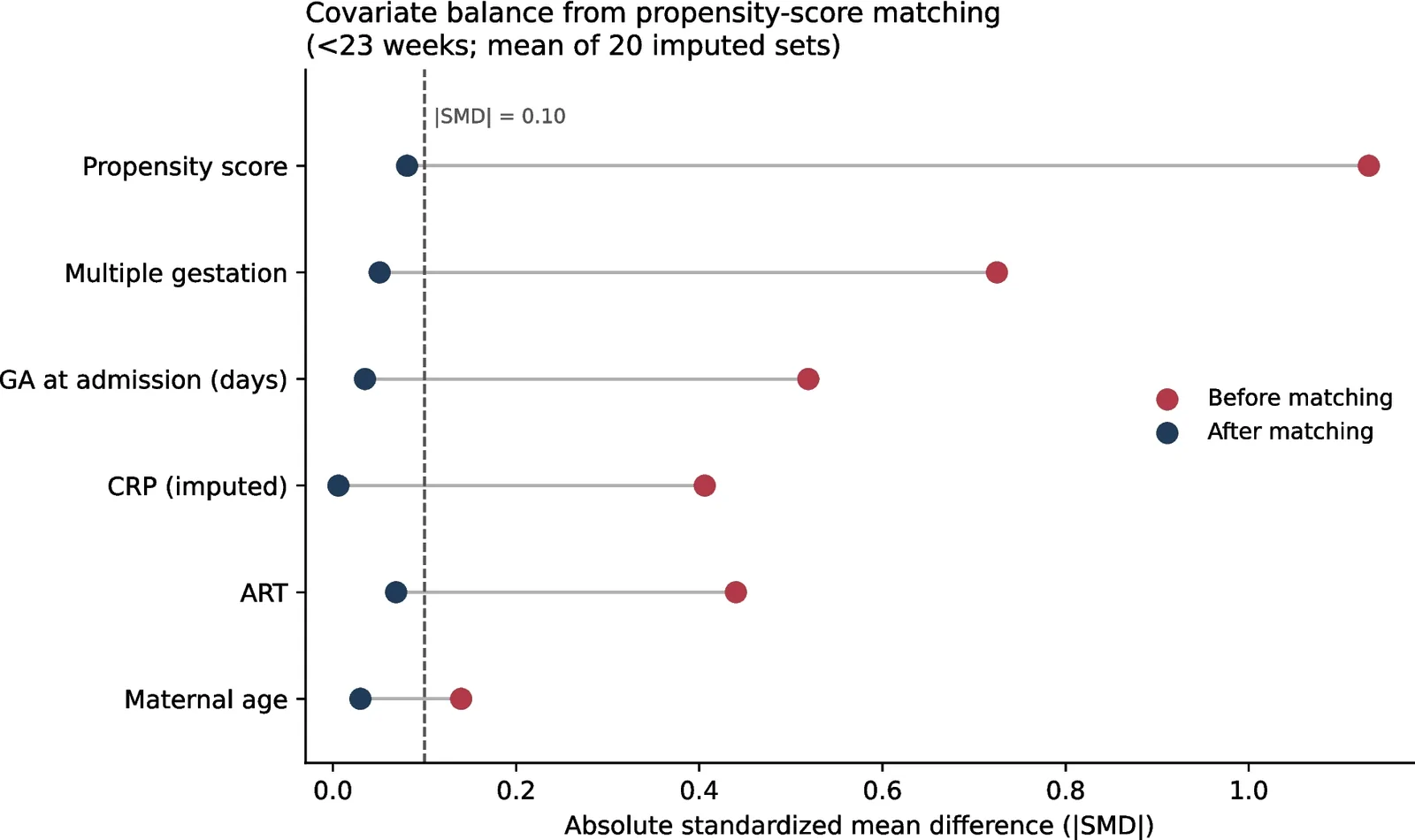
Covariate balance before and after propensity-score matching in the sub-23-week subcohort. Absolute standardized mean differences (|SMD|) for each covariate entering the propensity-score model, shown before matching (red) and after 1:1 nearest-neighbor matching within a 0.2-SD caliper (dark blue), averaged over 20 multiply imputed datasets. The dashed line marks the conventional |SMD|= 0.10 balance threshold. All covariates fell below 0.10 after matching (maximum 0.069, in ART conception), indicating adequate balance. ART assisted reproductive technology; CRP, C-reactive protein (multiply imputed); GA, gestational age; SMD, standardized mean difference

After propensity-score matching within the sub-23-week subcohort, all covariates were well balanced, with every covariate standardized mean difference below 0.10 (maximum 0.069; Table 4, Fig. 2). In the matched cohort, expectant management was independently associated with composite maternal morbidity (matched odds ratio 3.22, 95% CI 1.31–7.92, p = 0.011), with an absolute risk difference of 22.3% (95% CI 4.5–40.0; Table 5).

**Table 4:** Propensity-score matching: covariate balance and matched effect of expectant management on composite maternal morbidity (< 23-week subcohort). Covariate balance (mean of 20 imputed sets)

Covariates	|SMD| before	|SMD| after	< 0.10
Propensity score (logit)	1.131	0.081	Yes
Maternal age (years)	0.140	0.030	Yes
GA at admission (days)	0.519	0.035	Yes
Multiple gestation	0.725	0.051	Yes
ART conception	0.440	0.069	Yes
CRP (imputed, mg/L)	0.406	0.006	Yes

SMD, standardized mean difference. Matching: 1:1 nearest-neighbor, caliper 0.2 × SD of the logit propensity score (std.caliper = TRUE), without replacement. Mean matched EM cases: 46.5 of 64 [range 44–50] (≈18 EM off common support owing to the multiple-gestation positivity constraint). Maximum post-matching covariate |SMD|= 0.069 (ART); all covariate |SMD|< 0.10

**Table 5:** Matched composite maternal morbidity: expectant management versus termination

Estimates	Value (95% CI)	*p*
Matched Firth OR (EM vs TOP)	3.22 (1.31–7.92)	**0.011**
Matched risk difference	+ 22.3% (4.5 to 40.0)	—

Primary inference by Firth-penalized conditional logistic regression (subclass strata); risk difference by paired bootstrap (2000 resamples). CRP handled by multiple imputation (MICE, m = 20; outcome included), pooled by Rubin rules (FMI≈0.20). Sensitivity analyses are directionally consistent; with replacement, the CI widens as expected. Prolonged latency (expectant management) was associated with higher odds of CMM in the matched cohort

When latency was modeled directly (Tables 6 and 7), each additional day was associated with higher odds of composite maternal morbidity in the univariable analysis (odds ratio 1.014 per day, 95% CI 1.005–1.023, *p* = 0.003), but this association was attenuated after adjustment for management (odds ratio 1.008 per day, 95% CI 0.996–1.020, p = 0.209) and was absent within the expectant-management arm itself (odds ratio 1.006 per day, 95% CI 0.995–1.017, *p* = 0.299). Latency is strongly correlated with the management decision, so much of its apparent daily effect operates through management rather than through a progressive day-by-day accrual of risk. In the whole-cohort model, low-grade temperature elevation (> 37 °C) at admission was the strongest independent predictor of composite maternal morbidity (odds ratio 4.37, 95% CI 1.59–11.99, *p* = 0.005).

**Table 6:** Firth-penalized logistic regression for composite maternal morbidity and the daily effect of latency. Multivariable model (whole cohort, *n* = 211; 42 events)

Variables	Univariable OR (95% CI)	*p*	Multivariable OR (95% CI)	*p*
Expectant management (vs termination)	3.47 (1.75–7.12)	** < 0.001**	2.08 (0.76–5.70)	0.153
Gestational age at PPROM (per week)	1.05 (0.94–1.18)	0.401	1.03 (0.90–1.19)	0.640
Latency (per day)	1.014 (1.005–1.023)	**0.003**	1.008 (0.996–1.020)	0.209
Multiple gestation	3.40 (1.27–8.84)	**0.016**	1.88 (0.63–5.46)	0.250
Temperature > 37 °C at admission	3.57 (1.39–8.97)	**0.009**	4.37 (1.59–11.99)	**0.005**
AFI: oligohydramnios (vs anhydramnios)	2.27 (0.98–5.39)	0.056	1.44 (0.56–3.71)	0.445
AFI: normal (vs anhydramnios)	2.26 (0.99–5.33)	0.053	1.35 (0.50–3.66)	0.550

Firth-penalized likelihood (logistf); CIs by profile-penalized likelihood. Events per variable ≈6 (< 10), justifying penalization. All VIF < 2.2; no collinearity. Reference categories: termination, anhydramnios, singleton, ≤ 37 °C. One case with a missing admission temperature (> 37 °C) was excluded, so the model was fitted on 211 of 212 pregnancies (42 events). Significant p (≤ 0.05) in bold

**Table 7:** Daily effect of latency on composite maternal morbidity (model comparison)

Models	Latency OR per day (95% CI)	p
Univariable (whole cohort)	1.014 (1.005–1.023)	**0.003**
Multivariable (whole cohort, management included)	1.008 (0.996–1.020)	0.209
EM-internal (expectant only, 27 events)	1.006 (0.995–1.017)	0.299

Latency is significant univariably but not after adjustment for management, with which it is strongly correlated (r = 0.60); much of the daily latency effect operates through the management decision. The EM-internal model (expectant arm only) shows no residual within-arm daily latency effect. In the matched analysis (Table 4b), the management effect remains significant

Bold *p*-values indicate statistically significant results (*p*< 0.05)

**Fig. 3:**
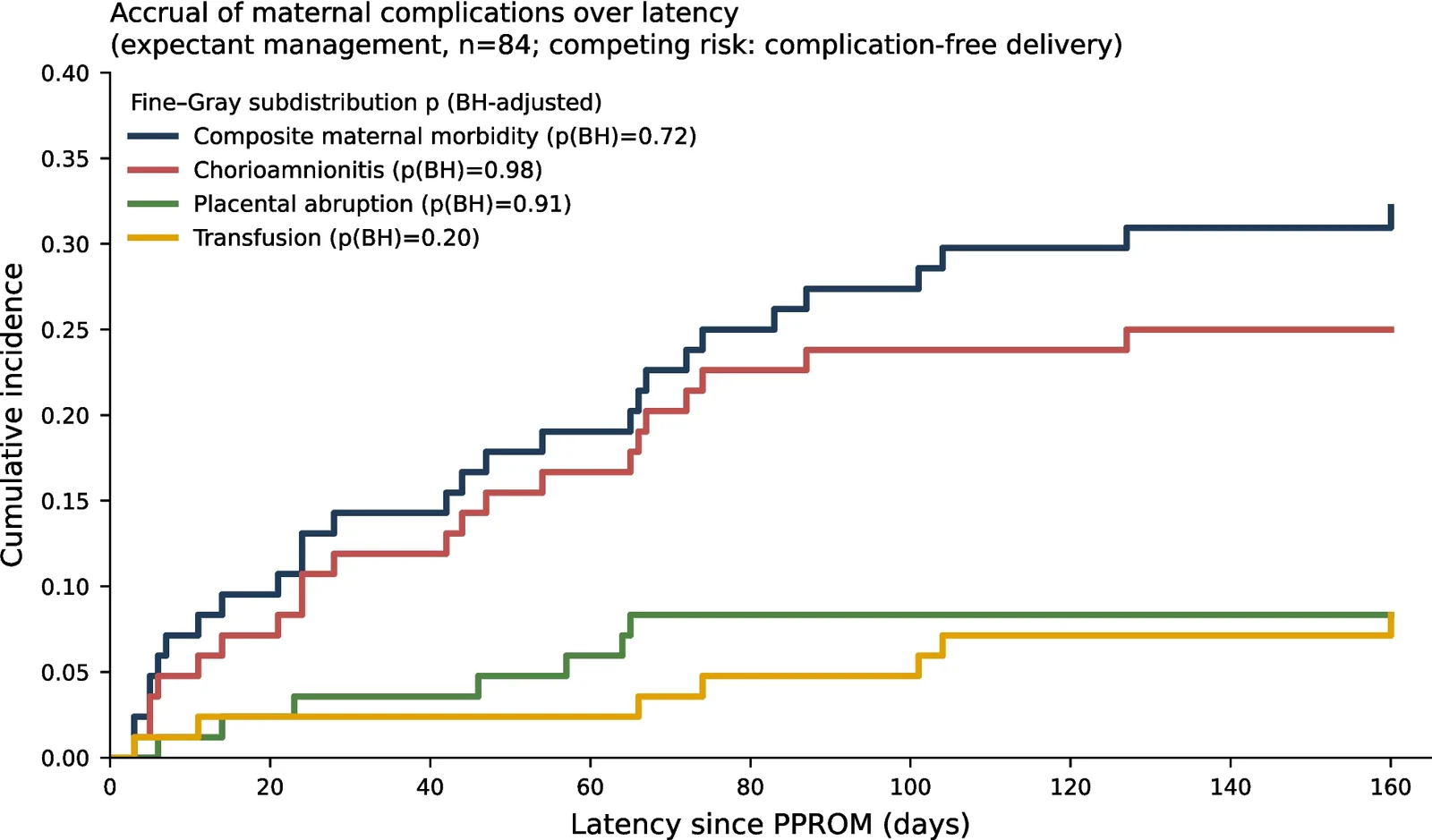
Cumulative incidence of maternal complications over latency under expectant management. Cumulative incidence functions for composite maternal morbidity and its components, with latency since PPROM as the time axis and complication-free delivery treated as the competing event (expectant-management cohort, n = 84). Curves are step functions of the Aalen–Johansen estimator. Legend p-values are Benjamini–Hochberg–adjusted Fine–Gray subdistribution p-values (family of five secondary time-to-event tests); none is significant after adjustment. The exact day of each complication was not recorded; patients were classified at delivery, so the curves describe accrual across latency rather than an instantaneous hazard. BH, Benjamini–Hochberg; PPROM, preterm premature rupture of membranes; sHR, subdistribution hazard ratio

In the competing-risks analysis of the expectant-management arm, maternal complications accrued gradually over the latency interval, with complication-free delivery treated as the competing event (Table 8, Fig. 3). No individual complication showed a significant subdistribution hazard after adjustment for multiple comparisons; the transfusion signal (raw p = 0.039) did not survive false-discovery-rate correction.

**Table 8:** Competing-risks analysis of maternal complications during expectant management (n = 84)

Outcomes (events)	CIF day 7	CIF day 120	Fine-Gray sHR/wk* (95% CI)	p (BH)	Cause-specific HR/wk (95% CI)
Composite maternal morbidity (27)	7.1%	29.8%	0.95 (0.85–1.07)	0.724	1.09 (0.94–1.27)
Chorioamnionitis (21)	4.8%	23.8%	1.00 (0.88–1.14)	0.980	1.15 (0.97–1.36)
Placental abruption (7)	1.2%	8.3%	1.10 (0.82–1.47)	0.905	—†
Transfusion (7)	1.2%	7.1%	0.84 (0.70–0.99)	0.197	—†

CIF, cumulative incidence function. Fine-Gray subdistribution and cause-specific Cox hazard ratios are *per gestational week; latency is the time axis and cannot enter as a covariate. p-values are Benjamini–Hochberg (BH) adjusted (family of five secondary time-to-event tests). †Cause-specific Cox only for outcomes with ≥ 20 events. Transfusion raw Fine-Gray p = 0.039 lost significance after BH adjustment (p = 0.197). Methodological limitation: the exact day of each complication was not recorded; patients were classified at delivery, so cumulative incidence describes accrual across latency without supporting a conventional instantaneous-hazard interpretation

**Fig. 4:**
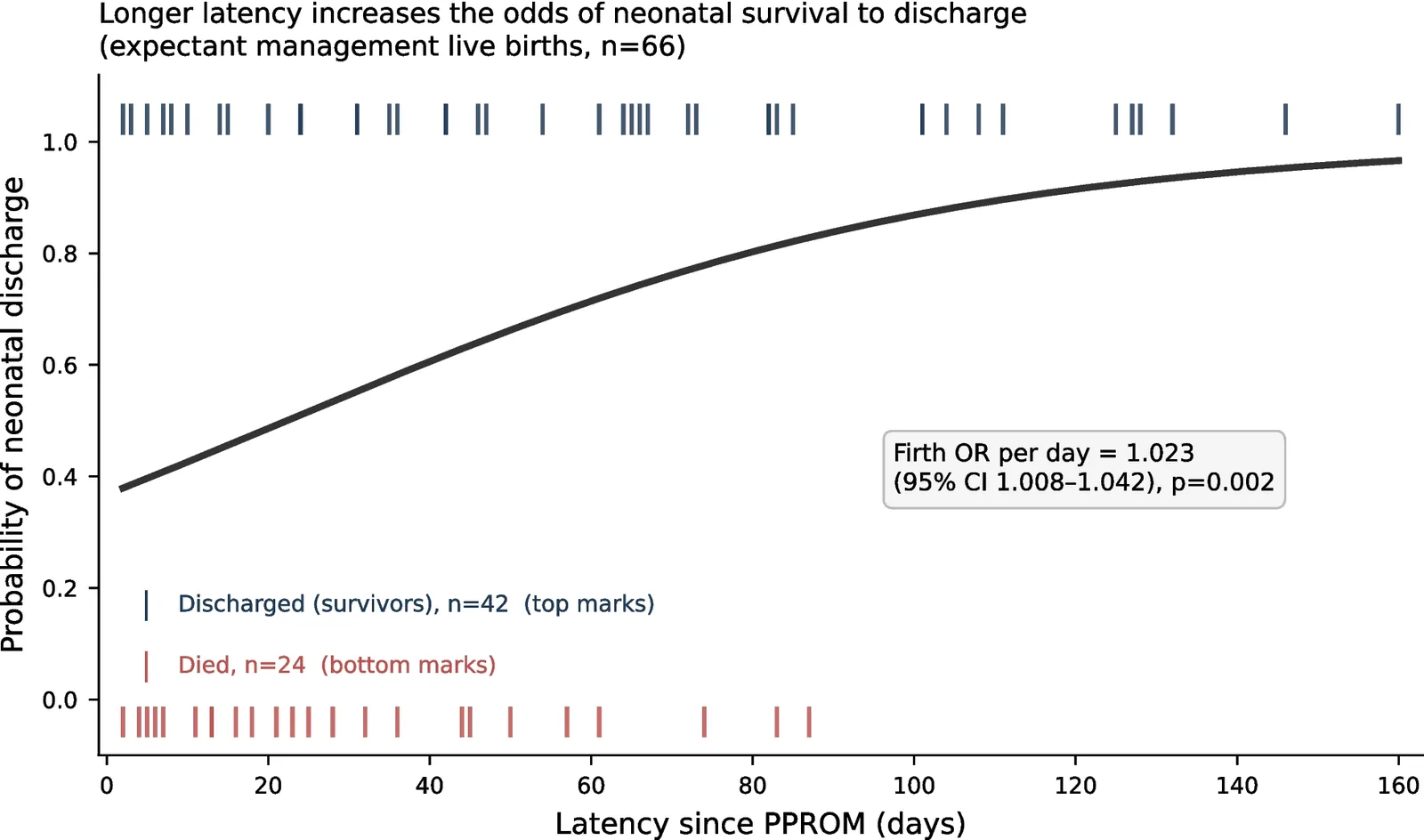
Longer latency is associated with a higher probability of neonatal survival to discharge. Predicted probability of neonatal discharge (survival) as a function of latency since PPROM among live-born infants in the expectant-management cohort (n = 66; 42 discharged, 24 died). The curve is a logistic fit; individual infants are shown as rug marks (discharged above, died below). The annotated estimate is the Firth penalized univariable odds ratio per additional day of latency (OR 1.023, 95% CI 1.008–1.042, p = 0.002). Firth penalization was used because of the limited number of events. Three live births with undocumented neonatal outcome were excluded. OR, odds ratio; PPROM, preterm premature rupture of membranes

In the multivariable Firth logistic regression model, both latency duration and gestational age at PPROM were independently associated with neonatal survival to discharge. After adjustment for gestational age at PPROM, the association between latency and survival became stronger rather than weaker (adjusted OR 1.058 per day, 95% CI 1.027–1.102, *p* < 0.001). Gestational age at PPROM was also an independent predictor of survival (adjusted OR 1.737 per week, 95% CI 1.216–2.720, *p* = 0.001). Table 9, Fig. 4). Gestational age at birth and birth weight were treated as intermediates on the causal pathway and were not used as adjustment covariates.

**Table 9:** Firth-penalized univariable logistic regression for neonatal survival to discharge (expectant-management live births, *n* = 66; 42 discharged, 24 died)

Predictors	OR (95% CI)	*p*
**Latency (per day)**	1.023 (1.008–1.042)	**0.002**
**Gestational age at PPROM (per week)**	1.019 (0.842–1.228)	0.844
**Gestational age at birth (per week)†**	1.499 (1.209–2.002)	** < 0.001**
**Birth weight (per gram)†**	1.002 (1.001–1.004)	** < 0.001**
Bivariable model (latency adjusted for gestational age at PPROM)		
**Predictors**	Adjusted OR (95% CI)	***p***
**Latency (per day)**	1.058 (1.027–1.102)	** < 0.001**
**Gestational age at PPROM (per week)**	1.737 (1.216–2.720)	**0.001**

Firth penalized-likelihood logistic regression with profile-likelihood 95% confidence intervals; fixed seed 2026. The bivariable model includes only latency and gestational age at PPROM. Gestational age at PPROM is measured before latency begins and is therefore a baseline confounder of the latency–survival relationship, not a mediator; adjusting for it yields the effect of latency independent of the gestational age at which membranes ruptured. †Gestational age at birth and birth weight are downstream consequences of latency (causal mediators) and are shown only as univariable associations; they are deliberately excluded from the bivariable model to avoid overadjustment bias (Schisterman 2009; VanderWeele 2009). With 24 events and 2 predictors, the events-per-variable ratio is ~ 12, and Firth penalization further guards against small-sample and separation bias. Bold denotes p ≤ 0.05. CI, confidence interval; OR, odds ratio; PPROM, preterm premature rupture of membranes

**Fig. 5:**
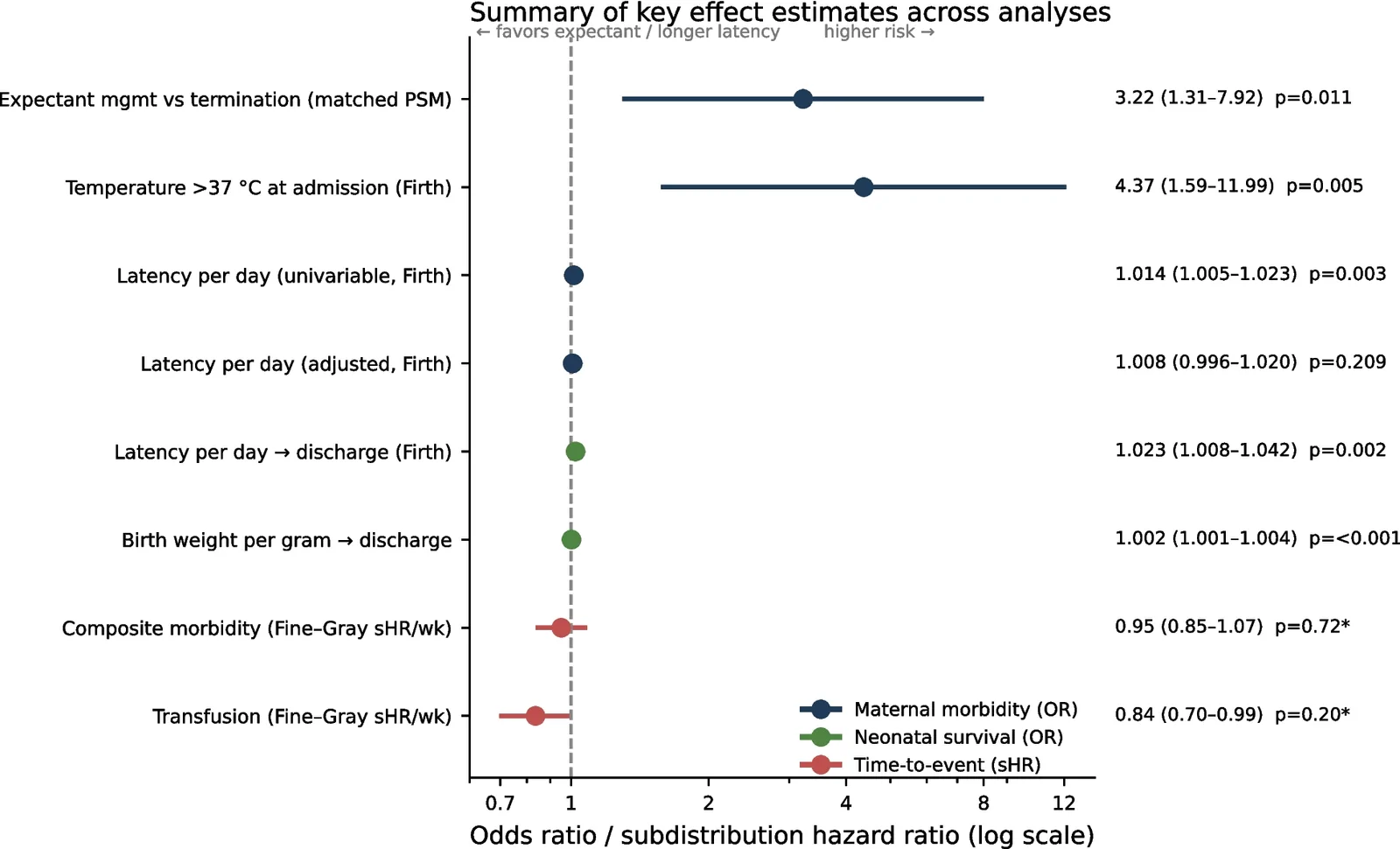
Summary of key effect estimates across the maternal and neonatal analyses. Forest plot of the principal effect estimates with 95% confidence intervals on a logarithmic scale, colored by analysis domain: maternal morbidity odds ratios (dark blue), neonatal survival odds ratios (green), and time-to-event subdistribution hazard ratios per week (red). The dashed line marks the null value of 1.0; estimates to its right indicate higher risk. Maternal-morbidity and neonatal odds ratios are Firth-penalized; the matched estimate derives from propensity-score matching. Time-to-event estimates are Benjamini–Hochberg–adjusted (asterisk). OR, odds ratio; PSM, propensity-score matching; sHR, subdistribution hazard ratio

The decomposition of the composite outcome and the supporting subgroup and sensitivity analyses were directionally consistent with the primary results (Supplementary Tables S1–S3). Estimates resting on sparse cells or lying at the significance boundary, including the multiple-gestation association with neonatal survival, were interpreted cautiously and descriptively. A summary of the principal effect estimates across analyses is provided in Fig. 5.

## Discussion

Our study characterizes the maternal risk profile in periviable PPROM and provides evidence of a stable risk–benefit balance during the latency period. While expectant management is associated with a more than three-fold increase in the baseline risk of CMM compared to termination (Matched OR 3.22; 95% CI 1.31–7.92), multivariate analysis demonstrates that this risk does not escalate on a daily basis (*p* = 0.299). Crucially, each additional day of pregnancy prolongation significantly enhances the probability of neonatal survival to discharge (Adjusted OR 1.058; 95% CI 1.027–1.102) without a corresponding linear increase in maternal complications. These findings suggest that under vigilant clinical supervision, the potential for improved fetal maturity may justify the baseline maternal risks, positioning expectant management as a reasonable clinical option to be discussed during shared decision-making in this vulnerable population.

In EPIPAGE-2 cohort including 379 pregnancies with PPROM between 22 and 25 weeks, no relationship was found between gestational age at PPROM and latency period, wheras higher gestational age at PROM was associated with later delivery [4]. Overall, 51.7% of fetuses survived to discharge. Neonatal outcomes varied by gestational age at PPROM, but rates of discharge without severe morbidity were similar across gestational ages. A prospective cohort of 884 infants born at 23–27 weeks due to PPROM, a latency period ≥ 7 days was not associated with improved neonatal survival [14]. Melamed et al. reported higher adverse neonatal outcomes in PPROM compared with spontaneous preterm birth at 28–33 weeks (53.7% vs. 42.0%; *P* < 0.001). In the absence of associated maternal complications, neonatal outcomes improved with longer latency durations [15]. In our study, among periviable PPROM cases, each additional day of latency duration was found to significantly improve neonatal survival.

Neonatal survival in this cohort was 50.0%, aligning with the 50%–60.9% range reported for periviable PPROM [4–6, 16]. While Sorano et al. observed higher survival (81.1%) reflecting Japan’s active resuscitation policies, 77.8% of those survivors faced severe morbidity [17]. Conversely, the prospective PPROMEXIL-III cohort reported a lower survival of 26.7%. [18] Notably, while existing literature identifies GA at rupture as a primary determinant of survival [4, 9, 12] and associates longer latency with improved outcomes [16–19], our results uniquely found no such association with GA at rupture. Instead, we demonstrated that latency duration is the critical factor: each additional day in utero significantly increased survival probability OR 1.058 (%95 GA 1.027–1.102; p < 0.001). This underscores that for periviable cases, the duration of pregnancy prolongation contributes more to survival than the initial GA at membrane rupture.

A meta-analysis of singleton periviable PPROM reported a 65.9% live birth rate with expectant management, a mean gestational age at delivery of 27.3 weeks, and a latency of 39.4 days. Chorioamnionitis occurred in 33.4% and maternal sepsis in 1.5%, with no maternal deaths [2].

A significant finding in our study was that pregnancies involving multiple gestations and those conceived via ART accounted for nearly a quarter (23.6%) of all cases that developed composite maternal morbidity**.** The incidence of PPROM is known to be higher in twins (7–10%) compared to singletons (2–4%), and multiple gestations are typically associated with a shorter duration from membrane rupture to delivery [25]. In line with this, contemporary evidence highlights that twin gestation is an independent risk factor for a significantly shorter median latency, often reported as 4 days compared to 7 days in singletons, as well as higher rates of neonatal sepsis and demişe [26]. While some studies suggest that expectant management can yield comparable perinatal outcomes in both singleton and twin pregnancies under standardized care [27], our cohort showed a trend toward reduced neonatal survival in multiples (OR 0.273, p = 0.059), which we interpret cautiously due to our limited sample size (n = 11).

This rising prevalence of multiple gestations in periviable PPROM is closely linked to the widespread use of assisted reproductive technology, which frequently results in multifetal pregnancies. However, the relationship between ART itself and neonatal outcomes remains complex. Some large-scale data suggest that intensive obstetric monitoring in ART singletons delivered before 28 weeks may actually reduce the risk of perinatal death (aRR 0.46) [23]; nevertheless, it is noted that these findings may be heavily influenced by selection bias and 'carryover effects' from parents who seek ART following previous pregnancy losses [24]. Ultimately, regardless of the mode of conception or plurality, latency duration remained the primary driver of survival in our study, with each additional day of pregnancy prolongation significantly increasing the odds of neonatal discharge (OR 1.023) [4].

In our cohort, prolonged anhydramnios was a primary indicator of clinical severity, with 6 out of 7 affected infants failing to survive. These results mirror findings from a similar cohort where anhydramnios was significantly more frequent in non-survivors (86.0% vs. 38.4%) and served as a key independent predictor of neonatal death [6]. While our initial analysis suggested a potential inverse association between anhydramnios and maternal morbidity (OR 0.451), we interpret this as selection bias rather than a biological benefit, as anhydramnios was more frequent in women electing pregnancy termination**.** Clinically, severe fluid loss during critical developmental windows is known to increase the risk of serious respiratory morbidity four-fold [28]. Given that low fluid levels complicate nearly half (47.1%) of periviable PPROM cases globally [2], its presence remains a cornerstone for counseling families regarding survival and the high risk of pulmonary hypoplasia [29].

Our clinical outcomes for expectantly managed periviable PPROM compare favorably with recent international benchmarks. Specifically, our 82% live birth rate and 43.4-day mean latency exceed the 65.9% survival and 39.4-day latency reported in a 2024 meta-analysis [2]. Despite this extended pregnancy prolongation, maternal infectious risks remained well-controlled; our incidence of clinical chorioamnionitis (25.0%) was lower than the 33.4% pooled average in the literatüre [2]. While maternal sepsis has been reported in up to 1.5% of periviable cases globally and even higher in some specific cohorts [13], we observed only one case (1.2%)Furthermore, our 8.3% rate of placental abruption aligns with rates documented in other modern series [4, 17]. These results suggest that close inpatient monitoring and standardized antibiotic prophylaxis for all PPROM cases effectively supported fetal maturation while maintaining high standards of maternal safety [3]. In a 15-year study of PPROM-related maternal deaths, 6 of 7 maternal deaths were due to sepsis and 1 to postpartum hemorrhage secondary to placenta accreta [20].

One maternal death reported in our study. The patient had a twin IVF pregnancy at 18 + 3 weeks with advanced cervical dilation and prolapsed amniotic sac. After PPROM, she opted for expectant management; spontaneous abortion occurred 2 days later. Following curettage for retained placenta, she developed DIC requiring massive transfusion, and subsequently died. Further investigations were not performed due to lack of consent.

Maternal morbidity rates in our cohort—32.1% for expectant management and 11.7% for termination—were notably lower than the 60.2% and 33.0% reported in a recent multicenter study. This difference is primarily explained by our more restrictive definition of composite maternal morbidity (CMM). Unlike Sklar et al., we deliberately excluded D&C from our primary outcome. In our clinical practice, revision curettage for terminations before 20 weeks is frequently performed as a discretionary procedure rather than as a mandatory response to retained tissue or infection. Given that D&C was performed in 78.9% of our termination cases, its inclusion would have systematically skewed the results and introduced a significant selection bias against the termination group. By focusing on objective clinical indicators, such as clinical chorioamnionitis and postpartum hemorrhage, we aimed to provide a more accurate assessment of the inherent risks of pregnancy prolongation. Even with these exclusions, our results confirm that expectant management remains independently associated with more than a three-fold increase in the odds of maternal morbidity (OR 3.22; 95% CI 1.31–7.92) [1].

In Sklar et al.’s study, the overall rate of live-born infants discharged home was 38.9% (15.7% without maternal morbidity, 23.2% with maternal morbidity) [1]. Two studies comparing active and expectant management in previable PPROM reported similar incidence of postpartum hemorrhage and maternal sepsis between groups [1, 21]. In our study, 28.5% of cases discharged a live infant without maternal morbidity, while 21.4% discharged a live infant despite experiencing maternal morbidity. The inclusion of PROM cases up to 24 weeks and 6 days in our study may explain this higher rate. In our clinic, all PPROM cases are admitted, closely monitored, and receive antibiotics, with prompt delivery if chorioamnionitis develops, which may account for the lower rates of sepsis and infectious complications compared with previous reports.

The only meta-analysis directly comparing maternal outcomes in previable PPROM emphasized the lack of data and studies regarding maternal outcomes [22]. It highlighted the need for further research comparing maternal health in cases managed both actively and expectantly. The study also reiterated the importance of appropriately informing the patient during the decision-making process.

Beyond immediate management strategies, identifying pregnancies at high risk for PPROM during the first trimester could significantly refine clinical care. Recent evidence from a large cohort suggests that low levels of PAPP-A (< 0.71 MoM) and free β-hCG during early screening (11–13 + 6 weeks) are associated with a three-fold increase in the risk of PPROM and spontaneous preterm birth. While these biochemical markers currently show limited individual predictive power (AUC < 0.7), integrating such early-stage risk markers with our findings on maternal and neonatal safety could facilitate a more preemptive counseling model [30].

### Strengths and limitations

A primary strength of this study is the relatively large cohort specifically evaluating periviable maternal morbidity, offering paired maternal-neonatal data to characterize complex clinical trade-offs. Data integrity was ensured through manual cross-verification of electronic and paper records. Crucially, we provide a novel perspective by demonstrating that maternal risk does not independently accumulate in a progressive, day-by-day manner during the latency period, adding vital evidence to the counseling framework.

Limitations include the retrospective, single-center design, which reflects institutional protocols. While we used propensity-score matching to address baseline imbalances, this method only accounts for measured factors; unrecorded clinical judgments regarding case severity may still influence outcomes. Additionally, because roughly one-quarter of expectant management cases could not be matched, the generalizability of those specific estimates is constrained. A structural limitation is that the exact timing of each maternal complication was not recorded, meaning our time-to-event data describe the pattern of risk accrual over latency rather than precise event times. Furthermore, certain rare and severe complications—including ICU admission, venous thromboembolism, and acute kidney injury—were not observed in our study population. Consequently, these events were not coded in our database, which technically prevented the construction of a full "severe maternal morbidity" subscore as defined by Sklar et al. Finally, while our multivariate model showed high specificity, its limited sensitivity may not capture all patients who experience complications, and the small sample size for subgroups like twin pregnancies (n = 11) necessitates a cautious, descriptive interpretation of those findings.

## Conclusion

In conclusion, while expectant management of periviable PPROM carries a three-fold higher baseline risk of maternal morbidity compared to termination, our findings demonstrate that this danger does not accumulate on a daily basis. Conversely, each additional day of pregnancy prolongation significantly enhances the probability of neonatal survival to discharge. These results indicate that provided there is rigorous medical supervision and an absence of acute complications, expectant management is a reasonable clinical option to optimize fetal outcomes without exposing the mother to a progressively increasing daily risk. This stable balance between risk and benefit is the cornerstone of individualized counseling for families facing PPROM at the limits of viability.

## Supplementary Information

Supplementary material 1: Table S1. Decomposition of composite maternal morbidity and its association with latency (competing-risks framework). Table S2. Supporting analyses: assisted reproduction / multiple gestation and anhydramnios. Table S3. Sensitivity analysis: daily effect of latency on composite maternal morbidity within the overlapping gestational-age window.

## Data Availability

The datasets used and/or analysed during the current study are available from the corresponding author on reasonable request.

## Abbreviations

AFI: Amniotic fluid index
ART: Assisted reproductive technology
CI: Confidence interval
CMM: Composite maternal morbidity
CRP: C-reactive protein
DIC: Disseminated Intravascular Coagulation
EM: Expectant management
GA: Gestational age
ICU: Intensive Care Unit
IQR: Interquartile range
OR: Odds ratio
PPROM: Preterm premature rupture of membranes
PAMG-1: Placental Alpha Microglobulin-1
DVP: Deepest Vertical Pocket
NST: Non-Stress Tests
PSM: Propensity-score matching
sHR: Subdistribution hazard ratio
SMD: Standardized mean difference
TOP: Termination of pregnancy
WBC: White blood cell count
