# Supplementary material for: Maternal morbidity and neonatal survival after periviable PPROM: the impact of latency duration
Source: BMC Pregnancy Childbirth. 2026 Aug 7;26:1269. doi: 10.1186/s12884-026-09759-x (PMC13732971; doi:10.1186/s12884-026-09759-x)
Supplement: Supplementary file 1 [file 12884_2026_9759_MOESM1_ESM.docx]

**Supplementary Tables**

**Supplementary Table S1.** Decomposition of composite maternal morbidity and its association with latency (competing-risks framework).

| **Subcomposites** | **All** | **EM** | **TOP** | **CIF d120** | **Fine-Gray sHR/wk (95% CI)** | **p (BH)** |
| --- | --- | --- | --- | --- | --- | --- |
| **Infectious (chorioamnionitis or sepsis)** | 33 | 22 | 11 | 25.0% | 0.99 (0.87–1.12) | 0.980 |
| **Hemorrhagic–surgical (atony/transfusion/laparotomy/hysterectomy)** | 14 | 9 | 5 | 9.5% | 0.91 (0.76–1.10) | 0.795 |
| **Partial severe/invasive (death/hysterectomy/laparotomy/transfusion)** | 11 | 7 | 4 | 7.1% | 0.84 (0.70–0.99) | —† |

One maternal death (expectant management) is retained within the composite (co-occurring transfusion and sepsis). †The partial severe/invasive indicator is descriptive only; a full Sklar (2022) severe-maternal composite could not be constructed because several components (ICU admission, readmission, VTE, acute kidney injury, uterine rupture, endometritis) were not coded.

**Supplementary Table S2.** Supporting analyses: assisted reproduction / multiple gestation and anhydramnios.

| **Analysis** | **Effect (95% CI)** | **p** |
| --- | --- | --- |
| **Anhydramnios → CMM (whole cohort, Firth)** | OR 0.451 (0.208–0.922) | **0.029** |
| **ART → neonatal discharge (EM, univariable)*** | OR 0.414 (0.100–1.625) | 0.203 |
| **Multiple gestation → neonatal discharge (EM)*** | OR 0.252 (0.055–0.995) | **0.049** |

*Consistent with univariable results in Table 7. Anhydramnios appears inversely associated with CMM (OR<1); this reflects selection—anhydramnios was more frequent in the termination group and rarer in the expectant group—and must not be interpreted causally.

**Supplementary Table S3.** Sensitivity analysis: daily effect of latency on composite maternal morbidity within the overlapping gestational-age window.

| **Universe** | **n** | **CMM events** | **Latency OR/day (95% CI)** | **p** |
| --- | --- | --- | --- | --- |
| **<23-week main universe (n=183, univariable)** | 183 | 37 | 1.016 (1.006–1.026) | **0.001** |
| **20–23-week narrow window (n=74, univariable)** | 74 | 16 | 1.005 (0.987–1.021) | 0.572 |
| **20–23-week narrow window (multivariable)** | 74 | 16 | 0.996 (0.972–1.016) | 0.676 |

Within the narrow window of overlapping gestational age, the latency effect attenuates, indicating that the univariable latency–CMM association operates largely through differences in gestational-age distribution and supporting the mediation/collinearity interpretation of Table 5.
